# An Unusual IgE-Mediated Hypersensitivity: Two Case Reports of Paracetamol Allergy

**DOI:** 10.7759/cureus.42240

**Published:** 2023-07-21

**Authors:** Rita Limão, Amélia Spínola Santos, Maria Conceição Pereira Santos, Elisa Pedro, Anabela Lopes

**Affiliations:** 1 Immunoallergology Department, Centro Hospitalar Universitário Lisboa Norte, Lisboa, PRT; 2 Clinical Immunology Laboratory, Instituto de Medicina Molecular João Lobo Antunes, Lisboa, PRT

**Keywords:** skin test, paracetamol, ige, basophil activation test, allergy

## Abstract

Paracetamol is one of the most commonly used analgesic and antipyretic agents worldwide, attributed in part to its excellent safety profile when administered at recommended doses. Paracetamol allergy is not common, and the majority of the reactions are related to the pharmacological action of cyclooxygenase 1 inhibition. Selective and Immunoglobulin E (IgE)-mediated hypersensitivity reactions are rare. In this article, the authors report two cases of paracetamol allergy in which the mechanism of IgE-mediated hypersensitivity was demonstrated by positive skin tests and basophil activation tests. We highlight the relevance of identifying the mechanism underlying the reaction since patients with IgE-mediated paracetamol allergies will be able to tolerate non-steroidal anti-inflammatory drugs.

## Introduction

Paracetamol is one of the most commonly used analgesic and antipyretic agents worldwide, in part due to the paucity of its adverse effects when administered at recommended doses [1]. At therapeutic doses, paracetamol is a weak cyclooxygenase (COX) 1, COX-2, and COX-3 inhibitor, but it is not a non-steroidal anti-inflammatory drug (NSAID) [2-4]. Paracetamol allergy is not common, and most of the reported reactions are related to the pharmacological action of COX-1 inhibition [5]. Most reactions are documented by oral challenge tests [6-8], which do not elucidate the underlying pathophysiological mechanism that, in rare cases, has been shown to be IgE-mediated, with positive skin tests and/or detectable serum-specific IgE [9-10].

In this article, we present two rare cases of paracetamol allergy in which the mechanism of IgE-mediated hypersensitivity has been demonstrated and supported by in vivo and in vitro tests.

This data has been presented as a poster at the 40th annual meeting of the Sociedade Portuguesa de Alergologia e Imunologia Clínica in October 2019.

## Case presentation

**Figure 1:**
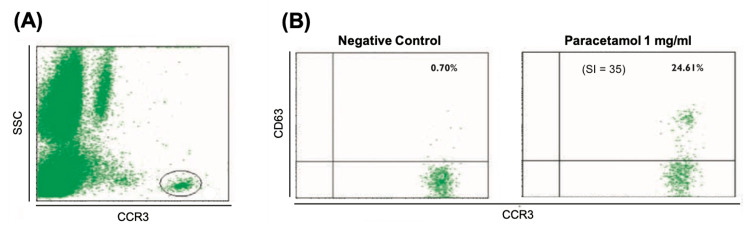
Basophil activation test for paracetamol: Case 1 SI: stimulation index (ratio of CD63% of activated cells to negative control).

The first case is a 30-year-old female with no history of drug reactions who presented her first episode of generalized urticaria and oropharyngeal tightness starting 10-20 minutes after oral administration of 1000mg of paracetamol (Ben-u-ron®) as a symptomatic treatment for headaches. A few months later, she reported a second and third episode of generalized urticaria within 10-15 minutes after oral administration of 250 mg of paracetamol, 20 mg of pyrilamine maleate, and 30 mg of caffeine (Antigrippine®) and 1000 mg of paracetamol (Ben-u-ron®), respectively, for symptomatic relief of headaches and coryza. There were no gastrointestinal, pulmonary, or cardiovascular symptoms in any episode. After the third episode, the patient was referred to an Immunoallergology appointment with no instructions to avoid any particular drug. The patient described prior tolerance to acetylsalicylic acid (ASA), ibuprofen, and nimesulide, which she maintained after the three episodes. The investigation of suspected immediate hypersensitivity to paracetamol was performed with skin prick tests (SPT) with injectable paracetamol solution (10 mg/ml) [11], with the appearance of a 4 mm mean diameter wheal (6.5 mm mean diameter wheal for histamine) and erythema with a larger diameter of 15 mm. A serum-specific IgE measurement for paracetamol using the ImmunoCap® method (Phadia, Thermo Fisher Scientific, Sweden) was specially requested and was negative. A basophil activation test (BAT) with a paracetamol solution (1 mg/mL) was also performed. This was positive, with a basophil activation rate of 24.61% (proportion of CD63+ cells) corrected for the negative control (0.70%) and a stimulation index (SI) of 35 (ratio of CD63% of activated cells and negative control) (Figure 1). This concentration did not induce any significant basophilic activation in four healthy individuals. The patient was advised to avoid paracetamol to avoid the recurrence of similar episodes.

**Figure 2:**
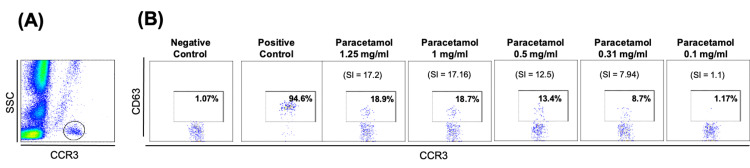
Basophil activation test for paracetamol: Case 2 (A) Identification of the basophil population as CCR3+ cells. (B) Flow cytometry dot plots of basophil activation expressed as a proportion of CD63+ on basophils corrected for the negative control. SI: stimulation index (ratio of CD63% of activated cells to negative control).

The second case is of a 26-year-old female who described her first episode of generalized urticaria after oral intake of 1000 mg of paracetamol (Doliprane®) and a second episode characterized by urticaria, and eyelid angioedema 10 minutes after oral administration of 1000 mg of paracetamol (Ben-u-ron®). A third episode of palmar and plantar itching, urticaria, and conjunctivitis started within 10 minutes after oral administration of 500 mg of paracetamol and 65 mg of caffeine (Ben-u-ron Caff®) for headaches. There were no signs or symptoms suggestive of pulmonary, gastrointestinal, or cardiovascular involvement in any episode. During the third episode, she was observed in the emergency department and treated with antihistamines and corticosteroids. She was referred to an outpatient Immunoallergology clinic with an indication to avoid paracetamol. After these three episodes, she tolerated ibuprofen, metamizole, and ASA. The immunoallergic research included SPT and intradermal tests with an intravenous paracetamol solution. The intradermal test at a concentration of 0.1 mg/ml (a non-irritant concentration as recommended by the European Network on Drug Allergy [11]) showed an immediate positive result, with an increase in the papule diameter of 3 mm and surrounding erythema of a greater diameter of 14 mm, at 15 minutes. According to the activation rate and the SI, Figure 2 shows a positive BAT for four paracetamol concentrations (0.31, 0.5, 1.0, and 1.25 mg/mL). The serum-specific IgE measurement for paracetamol was not performed.

Written informed consent was obtained from both patients, and they were treated according to the ethical standards established in the Declaration of Helsinki.

## Discussion

Hypersensitivity reactions to paracetamol are rare. Paracetamol is a weak COX inhibitor, and, therefore, inhibition of prostaglandin synthesis may be a mechanism to explain paracetamol reactions, similar to NSAIDs mechanisms [2-4]. However, cases of selective paracetamol reactions in NSAID-tolerant patients have been described [5]. In these cases, an IgE-mediated mechanism is possible.

In the few cases described of selective hypersensitivity to paracetamol, the diagnosis is mostly based on the oral challenge test, which does not elucidate the underlying pathophysiological mechanism [6-8]. The IgE-mediated mechanism was demonstrated by positive skin tests in only 10 reported cases [5,12-17] (Table 1).

**Table 1:** Reported cases of IgE mediated hypersensitivity to paracetamol ND: not done; NS: not stated; F: feminine.

Article	Patient	Symptoms/signs	Skin prick test	Intradermal test	Oral challenge or accidental re-exposure
Martin JA et al., 1993[12]	F, Adult	Generalized urticaria, angioedema	Positive	ND	Positive
Sabbah A et al., 1997[13]	F, 39 years	Urticaria	Positive	NS	Positive
Galindo PA et al., 1998[14]	F, 20 years	Generalized urticaria, respiratory distress, wheezing	Negative	Positive	ND
Paramo B et al., 2000[15]	F, 21 years	Generalized urticaria, angioedema	Positive	ND	Positive
	F, 24 years	Angiedema, hypotension	Positive	ND	Positive
Rutkowski et al., 2012[5]	NS	Urticaria, facial angioedema, conjunctivitis, cough	Positive	ND	Positive
	NS	Urticaria, facial angioedema, conjunctivitis, cough	Positive	ND	Positive
	NS	Generalized urticaria, dyspnea	Negative	Positive	ND
Numata et al., 2016[16]	F, 20 years	Urticaria, dyspnea	Positive	ND	ND
Teles et al., 2021[17]	F, 9 years	Generalized urticaria, facial angioedema	Negative	Positive	Positive

The authors describe two clinical cases of IgE-mediated hypersensitivity to paracetamol. Both are clinically characterized by the occurrence of several immediate-onset reactions 10-15 minutes after paracetamol administration, reproducible with repeated exposure, with no involvement of other drugs or allergen ingestion.

Both patients tolerate several NSAIDs, including ASA, demonstrating that COX-1 inhibition is not involved in these reactions. Although it is a limitation of the study, we considered that the benefit/risk ratio did not justify performing oral challenge tests to paracetamol in these patients because despite being the diagnostic gold standard of drug allergy, they are time- and resource-intensive and expose the patient to the risk of severe reactions. NSAIDs are effective antipyretic and analgesic drugs, making them a valid alternative to paracetamol.

The underlying specific IgE-mediated mechanism was demonstrated in both patients using paracetamol-positive skin tests. We also performed a BAT, which was positive in both patients. This basophil activation pattern confirmed the significant in vitro basophil degranulation specifically induced by paracetamol, which in these patients suggested an IgE-mediated mechanism [18-20]. Serum-specific IgE for paracetamol measurement was negative in the first case and was not performed in the second case. This analysis is not used in routine clinical practice, and some authors have reported negative results in patients with a proven IgE-mediated mechanism, which indicates that it has very low sensitivity [14,15].

## Conclusions

The authors describe two cases of immediate hypersensitivity reactions to oral paracetamol, reproducible with repeated exposure, and tolerance to several NSAIDs, including strong COX-1 inhibitors. The presence of positive skin tests allowed us to presume an IgE-mediated mechanism. Additionally, it was possible to observe in vitro a significant basophilic degranulation specifically induced by paracetamol, which in these patients supports the assumed mechanism.

We propose that skin tests should be performed before oral challenge as paracetamol-specific IgE may play a role in this type of allergy, and those patients with an IgE-mediated paracetamol allergy will be able to tolerate NSAIDs.
